# Clinical outcome of periodontal regenerative therapy using collagen membrane and deproteinized bovine bone mineral: a 2.5-year follow-up study

**DOI:** 10.1186/s13104-017-2426-y

**Published:** 2017-02-17

**Authors:** Daisuke Irokawa, Takahiro Takeuchi, Katsuya Noda, Hiroaki Goto, Masahiro Egawa, Sachiyo Tomita, Hiroki Sugito, Masahiko Nikaido, Atsushi Saito

**Affiliations:** 1 Department of Periodontology, Tokyo Dental College, Tokyo, Japan; 2 Private Practice, Goto Dental Clinic, Tokyo, Japan; 3 Department of Endodontics and Clinical Cariology, Tokyo Dental College, Tokyo, Japan; 4 Private Practice, Nikaido Dental Clinic, Tokyo, Japan

**Keywords:** Periodontitis, Guided tissue regeneration, Bone grafting, Biomaterial

## Abstract

**Background:**

This study aimed to evaluate, longitudinally, the outcome of periodontal regenerative therapy using a deproteinized bovine bone mineral (DBBM) in combination with a collagen barrier (CB) for the treatment of intrabony defects.

**Results:**

Patients with chronic periodontitis who have completed initial periodontal therapy participated in this study. They had at least one 2- or 3-wall intrabony periodontal defect of ≥3 mm in depth. During surgery, defects were filled with DBBM and covered with CB. Ten patients completed 2.5-year reevaluation. At baseline, mean clinical attachment level (CAL) of the treated site was 8.0 mm and mean probing depth (PD) was 7.5 mm. Mean depth of intrabony component was 4.6 mm. Mean gains in CAL at 6 months and 2.5 years were 2.8 ± 1.0 and 1.4 ± 1.5 mm, respectively, both showing a significant improvement from baseline. CAL gains at 1 and 2.5 years were significantly reduced from that at 6 months. A significant improvement in PD was also noted: mean reductions in PD at 6 months and 2.5 years were 4.0 ± 0.8 and 3.2 ± 0.8 mm, respectively.

**Conclusions:**

The combination therapy using DBBM and CB yielded statistically significant effects such as CAL gain and PD reduction, up to 2.5 years in the treatment of intrabony defects. However, the trend for decrease in CAL gain over time calls for the need for careful maintenance care.

## Background

Periodontitis is an inflammatory disease of tooth supporting tissues induced by plaque biofilm [1] and also considered as a dysbiotic disease with an adverse effect on systemic health [2]. In the treatment of periodontitis, initial periodontal therapy consisting of plaque control and scaling and root planing is the fundamental non-surgical treatment. In cases of moderate to advanced periodontitis, surgical interventions are often necessary.

Guided tissue regeneration (GTR) is defined as ‘a surgical procedure with the goal of achieving new bone, cementum, and periodontal ligament attachment to a periodontally diseased tooth, using barrier devices or membranes to provide space maintenance, epithelial exclusion, and wound stabilization’ [3]. GTR has been successfully used for the regeneration of periodontal tissues for more than three decades [4]. For the treatment of intrabony defects (periodontal defect within the bone surrounded by one, two or three bony walls or combination of thereof), especially those with uncontained configuration (1 or 2 wall) or ≥3-mm width, combination GTR therapy is recommended [5]. Various bone graft materials can be used with barrier membranes [6, 7]. GTR using a deproteinized bovine bone mineral (DBBM) combined with a collagen barrier (CB) membrane has been reported to yield more gains in clinical attachment level (CAL) than flap operation alone [8, 9]. Furthermore, the combination therapy demonstrated a significantly more periodontal regeneration than each individual component [10, 11]. Although these GTR therapies yielded clinically favourable results, a substantial degree of variability in the outcome has been reported with a marked center effect [6, 12, 13], and longitudinal data are still limited. Furthermore, according to the practice guideline by the Japanese Society of Periodontology, the evidence for added benefit of the combination GTR therapies is not yet sufficient [14].

Previously, we conducted a series of prospective multicenter studies of the GTR using a DBBM (Bio-Oss®, Geistlich Pharma AG, Switzerland) and a non-cross-linked CB (Bio-Gide®, Geistlich) in the treatment of intrabony defect, and reported that the combined use of the DBBM and CB yielded a significant gain in CAL and reduction in probing depth (PD) at 6 months following surgery [15, 16]. We hypothesized that the favourable clinical outcome can be sustained for longer period of time, following this combination therapy.

This study aimed to assess 2.5-year clinical outcome of periodontal regenerative therapy using DBBM in combination with CB in the treatment of intrabony defects in patients with chronic periodontitis.

## Methods

### Study design and participants

This study presents a subset of patients who were treated at Tokyo Dental College Chiba Hospital, Chiba, Japan as part of a multicenter study [16]. The original study was conducted as a prospective non comparative 6-month study, performed at five centers; four private practices specialized in periodontics in Tokyo, Japan, and one dental school clinic. The participants were recruited from patients with chronic periodontitis [17] from April 2013 to February 2014. This study was performed in accordance with the Helsinki Declaration, and the protocol was approved by the ethics committee of Tokyo Dental College (No.431). Written informed consent was obtained from all participants.

### Inclusion and exclusion criteria

Inclusion criteria consisted of having at least one 2- or 3-wall intrabony defect ≥3 mm in depth in interproximal area of teeth, interproximal sites with probing depth (PD) ≥6 mm, keratinized gingiva ≥2 mm, and good level of oral hygiene [mean plaque index (PlI) ≤1] [18]. Participants must have completed initial periodontal therapy consisting of plaque control and consecutive sessions of quadrant scaling and root planing within 3 months.

Systemic exclusion criteria were the presence of uncontrolled systemic diseases, smokers, allergy to collagen, concurrent or previous bisphosphonate, high dose corticosteroid or other drug therapy, current pregnancy or lactation and general contraindications for dental and/or surgical treatment. Those under 20 years old or with furcation involvements at target teeth are also excluded.

### Clinical examination

The following parameters were recorded by trained, calibrated examiners at baseline: after recording of gingival index (GI) [19] and PlI, PD and gingival recession (GR) were recorded in 0.5 mm increments using a pressure-sensitive periodontal probe (Gram Probe #2, YDM, Higashi Matsuyama, Japan). CAL was calculated as the sum of PD and GR. Bleeding on probing (BOP) was recorded as the presence or absence of bleeding following measurement of PD. Tooth mobility (TM) [20] was also recorded. Reevaluations were performed at 6 months, 1 and 2.5 years after surgery.

### Radiographic assessment

Semi-standardized radiographs were taken with the long cone paralleling technique using film holders (CID-3, Hanshin Technical Laboratory, Nishinomiya, Japan) with customized occlusal stents.

### Surgical procedures

The surgical intervention was implemented as described previously [16]. Briefly, following local infiltration anaesthesia, defects were accessed using the modified papilla preservation technique [21] or the simplified papilla preservation flap [22]. After removal of granulation tissue, scaling and root planning was performed. No root surface conditioning was used. Then the defects were filled with DBBM (Geistlich Bio-Oss®, 0.25–1 mm, Geistlich Pharma AG, Wolhusen, Switzerland), which had been pre-soaked in sterile saline. A porcine-derived non-cross-linked CB membrane (Geistlich Bio-Gide®, Geistlich Pharma AG) was then placed to cover the bone graft material and defect margin. No sutures were used to stabilize the CB. The flaps were then replaced to obtain full closure and sutured by modified vertical mattress and interrupted sutures using e-PTFE sutures (Gore-Tex® Suture CV-6, W.L. Gore and Associates, Flagstaff, AZ, USA). No periodontal dressing was used.

### Intrasurgical measurements

The following parameters were assessed after debridement of the area: (1) distance from the cemento-enamel junction (CEJ) to the bottom of the defect (CEJ-BD); (2) distance from the CEJ to the most coronal extension of the interdental bone crest (CEJ-BC). These measurements were performed at the deepest interdental point of the defect and recorded in 0.5 mm increments. The intrabony component of the defect (INTRA) was calculated as INTRA = (CEJ-BD) − (CEJ-BC).

### Postsurgical care

The patients received antimicrobial agents (typically cefdinir 300 mg/d, for 4 days). Standard analgesic was given as needed. Patients were instructed to rinse twice daily with an antimicrobial mouth rinse (Listerine® Fresh Mint, Johnson & Johnson, Tokyo, Japan) and to use the following oral hygiene procedures in the treated area for the first 4 postoperative weeks. The patients were asked to start gentle wiping of the operated dento-gingival area with an ultra-soft toothbrush from the third postoperative day. No interdental cleaning in the treated area was allowed during the first 4 weeks.

The sutures were removed after 14 days. Professional supragingival tooth cleaning was performed at weeks 1, 2 and 4. Thereafter, all patients were placed on the maintenance programs.

### Maintenance care

Following completion of the original 6-month study, the patients were assigned to maintenance or supportive periodontal therapy based on individual needs. They were asked to come in at 3-month intervals as appropriate. At these appointments, the patients’ oral hygiene status and periodontal conditions were evaluated and the oral hygiene procedures were reinforced as necessary. Scaling and professional tooth cleaning were performed as needed.

### Statistical analysis

At each visit, data were recorded in a case report form. After proofed for entry errors, the data were compiled by creating a computerized file. The primary outcome variable was the change in CAL. In the calculations, measurements at the same deepest point of the selected defect were included. CAL gain and PD reduction were also summarized as percentage changes from baseline values.

Repeated measures analysis of variance with Tukey–Kramer multiple comparisons test or Friedman test with Dunn’s multiple comparisons test was used to analyze changes in quantitative data over time. Comparisons for BOP data were made by Fisher’s exact test. Correlation between variables was analyzed by Spearman rank correlation. A software package (InStat version 3.10 for Windows, GraphPad Software, La Jolla, CA, USA) was used. A *p* value of less than 0.05 was considered statistically significant.

## Results

### Study participants and baseline clinical parameters

Out of 19 Japanese patients at Tokyo Dental College who have completed the original 6-month study [16], 10 patients (mean age 55.5 years old, 5 women and 5 men) were available for the follow-up 2.5 years post-treatment. The reasons for drop-outs were patient no-shows and appointment cancellations for transferring to a different dental clinic or for unknown reasons. Demographic information and clinical parameters at baseline are presented in Table 1.

**Table 1:** Patient demographics and clinical parameters at baseline

Variable	(n = 10)
Age (years; mean ± SD)	55.5 ± 14.8 (range, 28–76)
Sex (% women)	50
CAL (mm; mean ± SD)^a^	8.0 ± 1.2 (range, 6.0–10.0)
PD (mm; mean ± SD)^a^	7.5 ± 1.1 (range, 6.0–9.5)
GR (mm; mean ± SD)^a^	0.7 ± 0.9
PlI^a^	0.1 ± 0.1
GI^a^	0.6 ± 0.3
BOP positive (%)^a^	80
TM^b^	0.6 ± 0.5
Width of keratinized tissue (mm; mean ± SD)^b^	4.9 ± 1.7 (range, 3.0–8.0)
INTRA (mm; mean ± SD)^c^	4.6 ± 1.3 (range, 3.0–7.0)

*CAL* clinical attachment level, *PD* probing depth, *GR* gingival recession, *PlI* plaque index, *GI* gingival index, *BOP* bleeding on probing, *TM* tooth mobility, *INTRA* intrabony component

^a^Reference site

^b^Reference tooth

^c^Intrasurgical measurement

Each participant contributed one defect site. Treated teeth comprised 3 (30% of treated sites) incisors (2 maxillary, 1 mandibular), 1 (10%) canine (maxillary), 3 (30%) premolars (2 maxillary, 1 mandibular) and 3 (30%) molars (3 mandibular). The mean width of keratinized tissue was 4.9 mm. The mean value for INTRA was 4.6 mm.

### Change in clinical parameters

All participants had received systemic antimicrobial agents, analgesics and mouth rinse as instructed. The postoperative healing was generally uneventful. Throughout the study, none of the participants underwent any adverse event.

**Fig. 1:**
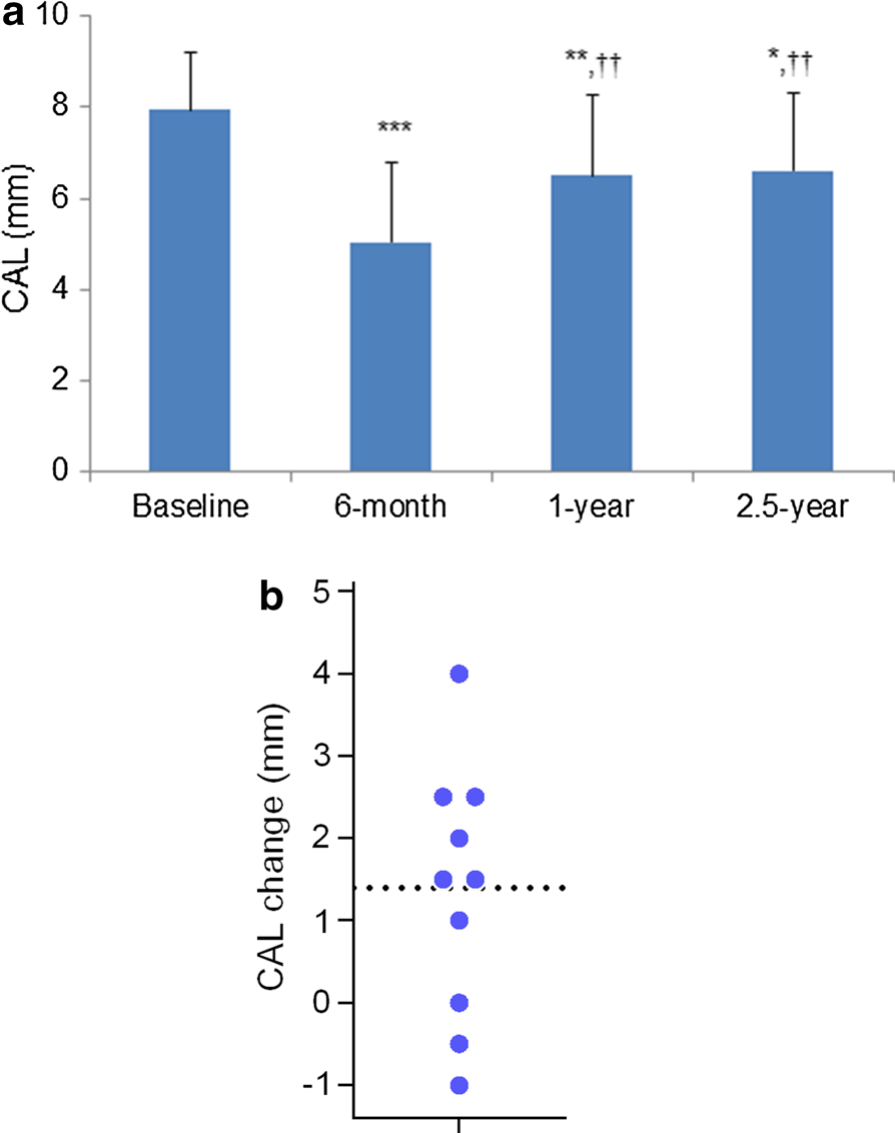
Change in CAL. **a** Mean CAL values at each evaluation period. Data shown as mean ± SD (n = 10). *P < 0.05, **P < 0.01, ***P < 0.001, compared to baseline, ^††^ *P* < 0.01, compared to 6 months, by ANOVA with Tukey–Kramer multiple comparisons test. **b** Distribution of individual CAL change from baseline at 2.5 years. *Dotted line* indicates the mean value.* CAL* clinical attachment level

At 6 months after surgery, a significant improvement in CAL from baseline was observed (*p* < 0.001) (Fig. 1a). A significant improvement from baseline was also observed at 1 year (*p* < 0.01) and at 2.5 years (*p* < 0.05). The CAL values at 1 and 2.5 years were significantly greater than that at 6 months (*p* < 0.01). The mean gains in CAL (primary outcome variable) at 6 months and 2.5 years were 2.8 ± 1.0 mm (range: 1.0 mm to 4.0 mm) and 1.4 ± 1.5 mm (range: −1 mm to 4.0 mm), respectively. Percentages of CAL gains at 6 months and 2.5 years were 35.5 and 17.0%, respectively.

Distribution of CAL change values at 2.5 years is shown in Fig. 1b. Seven participants showed CAL gains and three showed CAL losses or no change.

**Fig. 2:**
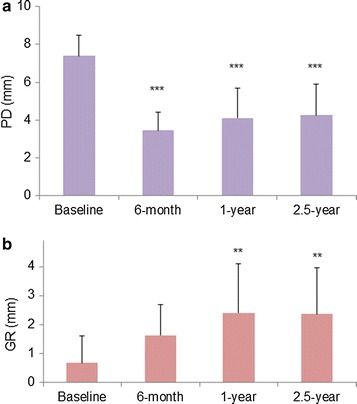
Change in PD (**a**) and GR (**b**). Data shown as mean ± SD (n = 10). **P < 0.01, ***P < 0.001, compared to baseline, by ANOVA with Tukey–Kramer multiple comparisons test. *PD* probing depth, *GR* gingival recession

**Fig. 3:**
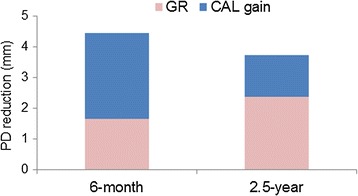
Contribution of GR and CAL gain to PD reduction. Mean values are shown. *GR* gingival recession, *CAL* clinical attachment level

As for the secondary outcome variables, a significant improvement in PD was noted at 6 months, 1 and 2.5 years (Fig. 2a). No significant differences in PD values were observed between 6 months, 1 and 2.5 years. The mean reductions in PD at 6 months and 2.5 years were 4.0 ± 0.8 and 3.2 ± 0.8, respectively. Percentages of PD reductions at 6 months and 2.5 years were 53.0 and 42.3%, respectively. There was a gradual increase in GR from baseline to 1 year after surgery (Fig. 2b). A significant difference from baseline in GR values was observed at 1 and 2.5 years (*p* < 0.01). However, no significant difference between 1 and 2.5 years was noted. Compared to 6 months, contribution of GR to the reduction in PD was greater at 2.5 years (Fig. 3).

As expected, baseline values for PlI, GI, and TM were relatively low, because the participants have already received initial periodontal therapy. A further, significant reduction in GI from baseline was found at 1 and 2.5 years (Table 2). No significant differences in PlI and TM were found during the observation periods. As for BOP, a significant difference from baseline was noted at 2.5 years (Table 3).

**Table 2:** Change in PlI, GI, and TM

Variable	Baseline	6-month	1-year	2.5-year
PlI	0.1 ± 0.1	0.2 ± 0.4	0.3 ± 0.3	0.3 ± 0.3
GI	0.6 ± 0.3	0.1 ± 0.2	0.03 ± 0.1**	0.1 ± 0.1**
TM	0.7 ± 0.5	0.4 ± 0.5	0.4 ± 0.5	0.5 ± 0.5

Data shown as the mean ± SD (n = 10)

*PlI* plaque index, GI gingival index, *TM* tooth mobility

** *P* < 0.01, significantly different from baseline, by Friedman test with Dunn’s multiple comparisons test

**Table 3:** Change in BOP

Variable	Baseline	6-month	1-year	2.5-year
BOP^a^	80	40	30	20*

*BOP* bleeding on probing

* *P* = 0.023, by Fisher’s exact test (two-sided)

^a^Percent sites positive

### Relationship between baseline variables and CAL gain or PD reduction at 2.5 years

No significant correlation was found between CAL gain at 2.5 years and baseline variables (Table 4). PD reduction at 2.5 years was significantly positively correlated with baseline PD and INTRA, indicating that the deeper the initial PD or INTRA, the greater tends to be PD reduction after surgery.

**Table 4:** Correlations between baseline valuables and CAL gain or PD reduction at 2.5 years

Baseline variable	CAL gain		PD reduction	
	*r*	*P*	*r*	*P*
CAL	0.341	0.330	−0.408	0.245
PD	−0.047	0.892	*−0.816*	*0.006*
TM	0.191	0.584	0.120	0.733
INTRA	0.273	0.448	*0.719*	*0.023*
Patient age	−0.207	0.560	0.114	0.759

*r*, Spearman coefficient. Significant differences are indicated in italics

*CAL* clinical attachment level, *PD* probing depth, *TM* tooth mobility, *INTRA* intrabony component

**Fig. 4:**
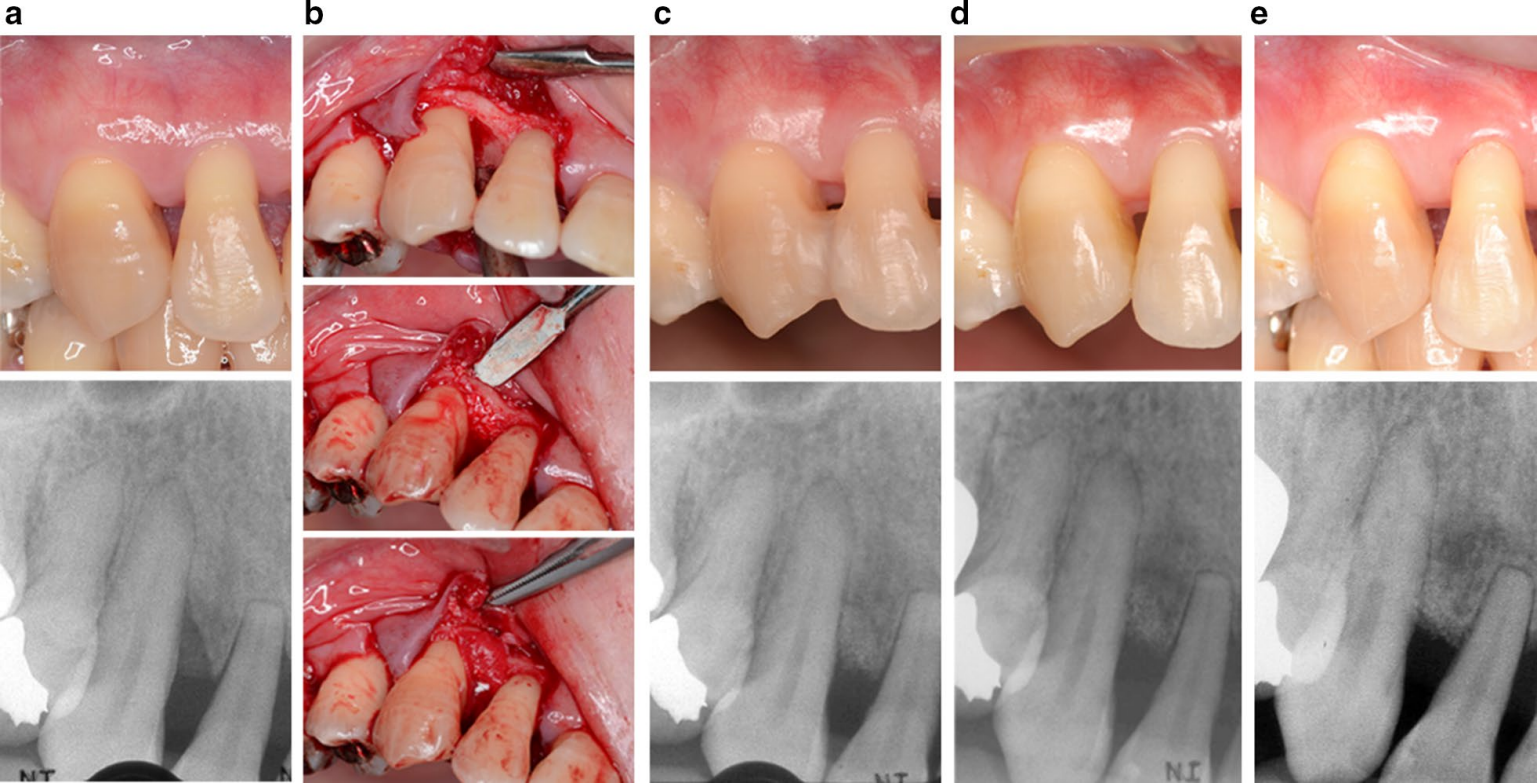
A representative treatment case. 42-year-old woman. **a** Before surgery (baseline), in the mesial aspect of the maxillary right canine; PD 6.0 mm, CAL 7.0 mm (*top*). In the radiograph, vertical bone defect is observed (*bottom*). **b** During surgery, careful scaling and root planing was performed after removal of granulation tissue (*top*). Then the defect was filled with DBBM (*middle*) and covered with CB (*bottom*). **c** Postoperative views at 6 months, **d** 1 year, **e** 2.5 years; PD 2.0 mm, CAL 3.0 mm (*top*). In the radiograph, an improvement in the initial bone defect area can be observed (*bottom*)

A representative case is shown in Fig. 4.

## Discussion

In the present study, we evaluated longitudinally the clinical outcome of periodontal regenerative therapy using DBBM in combination with CB in the treatment of intrabony defects in Japanese patients with chronic periodontitis. The combination therapy yielded statistically significant gains in CAL and reductions in PD at 2.5 years, when compared with the preoperative values. However, the trend for decrease in CAL gain over time was noted. These results should be interpreted with caution in terms of their clinical relevance.

A meta-analysis study has indicated that combination regenerative therapies performed better than the single therapies, although the additional benefits were small [23]. In the present study, significant PD reductions from baseline were sustained up to 2.5 years, and no significant difference in PD was observed between 6 months and 2.5 years. As for the CAL gain, the mean value was 1.4 ± 1.5 mm at 2.5 years after the combination therapy. The value was significantly smaller than that obtained at 6 months (2.8 ± 1.0 mm). Moreover, the CAL gain at 2.5 years is smaller than the values in the previous studies of combination regenerative therapies with longer observation periods. In a case-series of 15 patients, Stavropoulos and Karring [24] showed the mean CAL gain of 4.1 ± 1.3 mm at 5Y after the GTR therapy using the DBBM in combination with PLA/PGA bioabsorbable membrane. In their randomized clinical trial [25], the mean CAL gain of 2.3 ± 2.1 mm was noted in 10 patients at 6Y after the same combination therapy. In a randomized controlled study, Sculean et al. [26] demonstrated the CAL gain of 3.7 ± 1.1 mm in 10 patients at 5 years after the same combination therapy as the present study. The mean CAL gain in the present study was also smaller than the values observed in our previous studies of different regenerative therapies. In our 5-year clinical evaluation of GTR using a non-resorbable membrane only [27], the mean CAL gain was 3.6 mm, and another study using the enamel matrix derivative (EMD) showed the value of 3.4 mm at 2 years [28]. Even among studies using the same materials, it is difficult to directly compare clinical results because of the differences in the skill of surgeons, incision and flap designs and baseline CAL or PD values of the participants. For example, the baseline mean PD in the study by Sculean et al. [26] was 9.1 mm whereas the value for our study was 7.5 mm. It has been reported that a greater gain in CAL can be expected after regenerative therapy, in patients with greater initial values in PD and CAL [29, 30], although no significant correlation was found between the CAL gains at 2.5 years and baseline PD or CAL values in the present participants.

In the present study, two participants showed CAL losses at 2.5 years from baseline (−1.0 and −0.5 mm), although they both had CAL gains of ≥3.0 mm at 6 months. Given the small number of participants, these negative values contributed to the relatively modest mean CAL gains observed at 2.5 years. Moreover, at 2.5 years, seven out of 10 participants showed a decrease in CAL gain values from 6 months. It is notable that the mean PD of the participants at 6 months was 3.5 mm, which is somewhat greater than the value (2.7 mm) reported in our previous study using EMD [28]. These data may indicate the need for more meticulous postoperative maintenance care in the present participants. A partial loss of the CAL gain obtained 1 year after GTR has been shown to be associated with smoking, compromised oral hygiene [31], and poor compliance with a supportive periodontal program [32, 33]. All participants were asked to follow 3-month recall, but some (including those with CAL loss) failed to comply with this, resulting in longer maintenance intervals.

It has been shown that newly formed periodontal tissues in intrabony defects can be managed for ≥10 years [34]. It is unclear whether more frequent or meticulous supportive periodontal therapy could have prevented such CAL losses in the two patients. In a previous randomized clinical trial of the combination therapy, it was reported that poor oral hygiene, frequent BOP and infrequent maintenance visits did not seem to play a critical role in the long-term stability of the CAL gain [25]. However, patient compliance and appropriate periodontal maintenance are generally considered to be important for long-term success of periodontal regenerative therapy [5]. We continue to provide careful maintenance care to the participants in the present study.

There are several limitations of this study. First, the sample size was small and the data were from one institution. Second, the study was non-random and uncontrolled. A controlled, longitudinal study with larger sample size is needed to better evaluate the long-term performance of the combination therapy. Since periodontal disease is a multifactorial disease, the use of multivariate analysis would be more appropriate to analyse data from larger sample size. Also, it is not possible to confirm that periodontal regeneration had indeed occurred to the treated site, because no histological analysis can be presented. Lastly, the surgical procedures were performed by seven periodontists with various experience levels. This may have contributed to the differences in clinical outcomes among the participants, as indicated by the high standard deviations.

Despite these limitations, the study findings add significantly to the existing literature on the clinical effectiveness of the combination regenerative therapies. The participants in the present study are currently being followed up to evaluate more longitudinal treatment outcomes.

## Conclusions

The periodontal regenerative therapy using DBBM and CB produced significant clinical effects such as gain in CAL and reduction in PD, up to 2.5 years in the treatment of intrabony defects. There was a trend for decrease in CAL gain over time. Further studies are required to determine the true benefit of and appropriate case selection for this combination therapy. Such information may contribute to the optimization of the periodontal regenerative therapy.

## Abbreviations

DBBM: deproteinized bovine bone mineral
CB: collagen barrier
CAL: clinical attachment level
PD: probing depth
BOP: bleeding on probing
GI: gingival index
PlI: plaque index
TM: tooth mobility
GR: gingival recession
